# The intersection between human metapneumovirus and the respiratory microbiome

**DOI:** 10.1186/s12985-025-02872-x

**Published:** 2025-12-22

**Authors:** Oluwatoyin Modupe Aladejana, Damilola Feyisike Ayorinde

**Affiliations:** 1 Department of Biological Science, Redeemers University, Ede, Osun State Nigeria; 2 Department of Internal Medicine, Ekiti State University Teaching Hospital, Ado-Ekiti, Nigeria

**Keywords:** Human metapneumovirus, Microbiome, Next-generation sequencing, CRISPR-Cas12a, Probiotics

## Abstract

Human metapneumovirus is one of the viral causes of respiratory illness that can range from mild to life-threatening diseases. In December 2024, there was news about increased cases of human metapneumovirus (HMPV) in China, when 6.2% and 5.4% of positive respiratory illnesses and admissions, respectively, were linked to HMPV, surpassing adenovirus, rhinovirus, and COVID-19. There have been concerns about it becoming another epidemic, and by implication, a pandemic, especially as the world is gradually recovering from COVID-19 and its devastating impacts. Currently, there is no directly acting antiviral drug targeting HMPV, and this has left a gap in its treatment and management, especially in the young, elderly, and immunocompromised, who are prone to having severe manifestations. As the immune system is crucial in fighting and eliminating the infection, modulating the immune system directly or indirectly can treat HMPV. The lung that was initially known to be sterile is now found to house different populations of microorganisms, including bacteriome, virome, and mycobiome. The lung microbiome modulates HMPV infection. The presence of pathobionts like H. influenzae enhances HMPV infection and severity. The detection of the microbiome was made possible by the advent of cutting-edge technologies like next-generation sequencing and bioinformatics tools. The combination of Recombinase Polymerase Assay, CRISPR-Cas12a, and Fluorescence Assay has been used in the rapid detection of HMPV in China. The microbiome plays a crucial role in shaping the immune system. Exploring such can be a way of managing HMPV. Probiotics, prebiotics, and postbiotics are ways in which the microbiota can be manipulated to limit adverse drug reactions. These can be explored in HMPV diagnosis, treatment, and prevention.

## Introduction

Human metapneumovirus is one of the viral causes of respiratory illness that can present from mild to life-threatening diseases. Exactly five years after the onset of the last pandemic- the COVID-19 pandemic- the Chinese Center for Disease Control reported a sharp increase in respiratory infections, including Human metapneumovirus (HMPV) [1]. That HMPV causes increased morbidity and mortality in children compared to COVID-19 makes it a disease of concern [2, 3]. The epidemiological and Clinical Characteristics of Human Metapneumovirus-Associated Acute Respiratory Tract Infection Cases in the Pudong New Area, Shanghai, from 2014 to 2023 done by Ding et al., and published in March, 2025, revealed 1.77% HMPV infection rate, with cough in about 90–100% cases, with runny nose and sore throat being the most predominant clinical presentation especially in children, and expectoration seen more in adults [4]. According to the Epidemiology of human metapneumovirus among children with severe or very severe pneumonia in high pneumonia burden settings: the Pneumonia Etiology Research for Child Health (PERCH) study experience conducted in 2024 by Miyakawa et al., HMPV-associated severe paediatric pneumonia in high-burden settings was predominantly in young infants and clinically indistinguishable from RSV, as its case fatality was similar to that in RSV-positives [5]. Seemingly healthy adults who get infected can develop severe disease [6]. Currently, there is no vaccine for the disease. These show that more still needs to be known to prepare and prevent it from becoming an outbreak and eventually a pandemic.

**Fig. 1:**
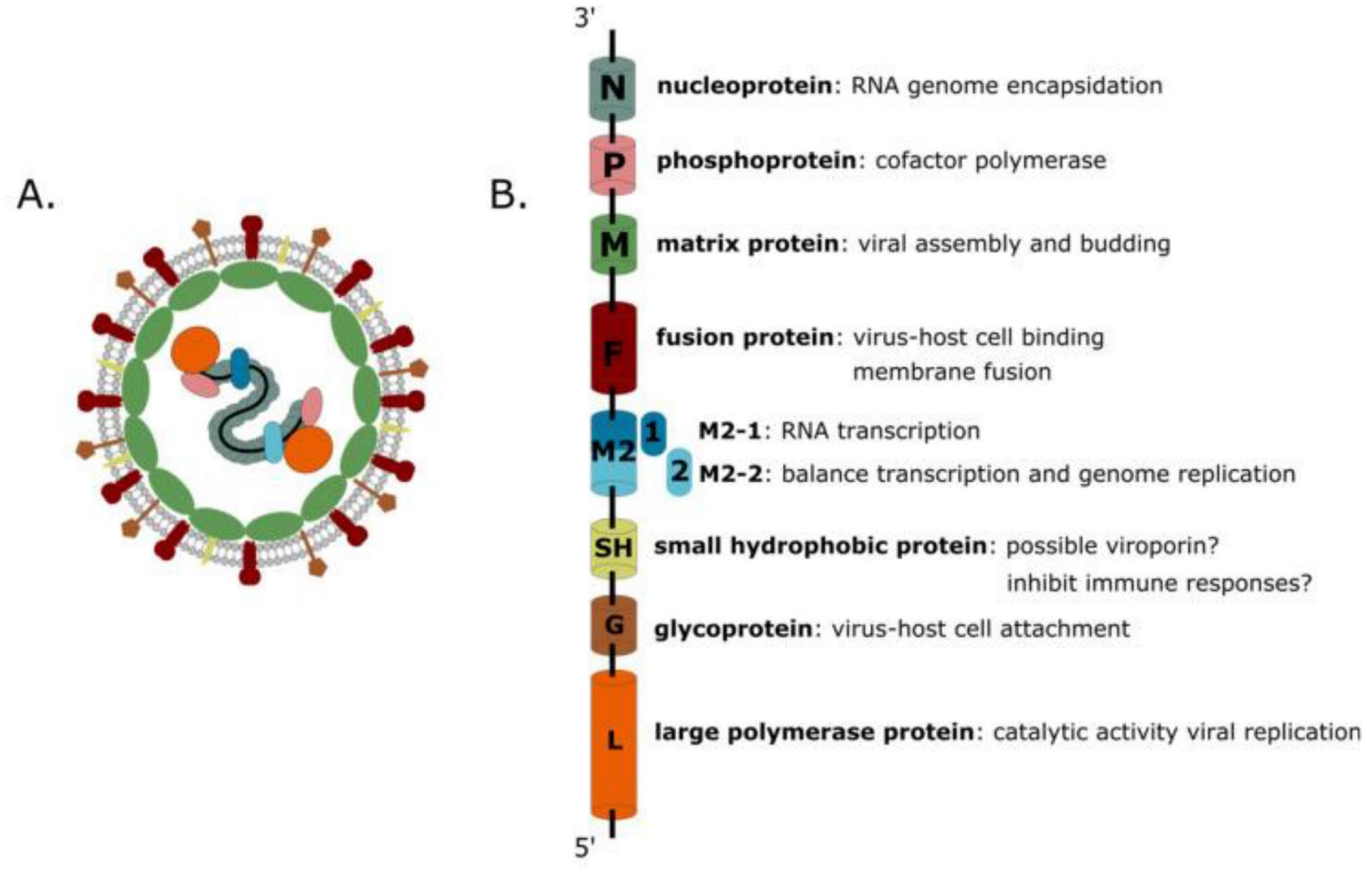
Protein encoded by HMPV RNA [7]

The human metapneumovirus was discovered in the Netherlands by Bernadette G. van den Hoogen in 2001 but has been circulating among humans for about 5 decades prior. It is a single-stranded, negative-sense RNA virus reclassified in 2016 as a *Pneumoviridae* of the order *Mononegavirales*. A human metapneumovirus particle is an RNA virus of about 200 nm in diameter. The RNA, about 13 kb long, is non-segmented, negative-sense and single-stranded encoding for nine proteins. These are Nucleoprotein (N), Phosphoprotein (P), Matrix protein (M1), Fusion protein (F), M2 (M2-1, M2-2), Small Hydrophobic protein (SH), Glycoprotein (G), and Large polymerase protein (L) as shown in Fig. 1 [7].

Human metapneumovirus (HMPV) primarily infects epithelial cells in the respiratory tract but can also infect alveolar macrophages and dendritic cells [7]. It spreads by inhalation of infectious droplets in the air, making contact with contaminated surfaces, or coming into close contact with infected persons. The spectrum of clinical presentation varies from mild symptoms of the upper respiratory tract, like fever, rhinorrhea, and cough, to severe ones involving the lower respiratory tract, e.g., wheezing, hypoxia and difficulty with breathing [8]. The radiological investigation of HMPV is usually by a chest computed tomography (CT) scan, which commonly reveals ground-glass opacity. The very young, elderly and immunocompromised tend to have severe symptoms with increased morbidity and mortality [9, 10]. Pregnant women who contract infection during pregnancy tend to deliver small- for gestational-age babies, too [11].

### The respiratory microbiome

**Fig. 2:**
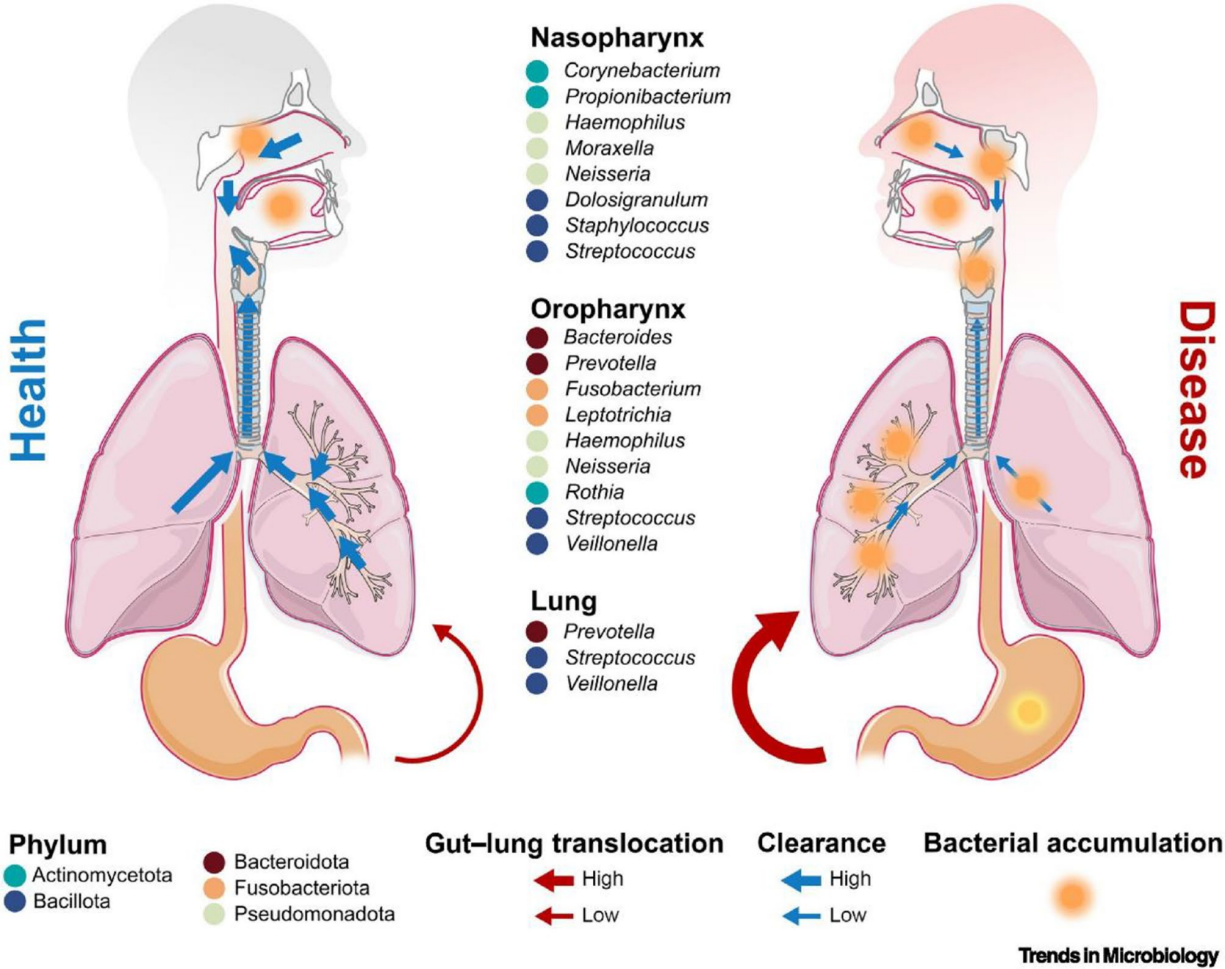
The Bacteriome of the Respiratory Tract [21]

The nostril is the beginning of the respiratory tract, and it ends in the lungs. The nose and the oropharynx are known to harbour different colonies of microorganisms, while the lower respiratory tract thought to be a sterile environment in a healthy individual has recently, through technological advancement, been proven otherwise [12]. The upper airway colonies were acquired early in life through several mechanisms, and those of the lungs were obtained from the upper respiratory tract [13]. The human nasal microbiome is less abundant than the gut’s. *Actinobacteria*, *Firmicutes* and *Proteobacteria* are commonly found [14]. The Lung microbiome is closely related to the oropharynx but not the nasal microbiome. The lung microbiomet is believed to arise from micro-aspirations that spread from there to the lungs. The oropharynx is house to numerous microbes, and the microbiome is closely related to oral and gastrointestinal diseases [15]. Organisms commonly found in the oropharynx include bacteria, viruses, protozoa, fungi and archaea [16]. The lung microbiota has bacteria, fungi, and viruses as its constituents. In healthy individuals, the lung bacteriome consists of Firmicutes, Proteobacteria and Bacteroidetes [17]. A common genus of bacteria found is *Streptococcus*, followed by *Prevotella*, *Fusobacterium*, and *Veillonella* [18]. These are as depicted in Fig. 2. The mycobiome in the lungs shapes the development of immunity in the respiratory system by preventing pathogenic organisms from attaching and priming the immune system. The mycobiome populations in the respiratory tract are *Eurotium*,* Claudosporium* and *Aspergillus* [19]. The viruses found as part of the respiratory virome *are Paramyxoviridae*,* Orthomyxoviridae*,* Picornaviridae*,* Adenoviridae*,* Polyomaviridae*, and *Redondoviridae* [20].

### Factors affecting the respiratory microbiome

The nasal microbiome is constantly changing due to its connection with the environment. The factors to be considered for the lower airways are acute or chronic oropharyngeal and respiratory illnesses, use of antibiotics, presence of a foreign body, use of probiotics, and immune status. Periodontal diseases can be a source of pathogenic organisms that spread to the lungs directly or alter the immune status enough to make the individual prone to respiratory infections [22]. Acute and chronic respiratory illnesses like COVID-19, human metapneumovirus, bacterial pneumonia, Chronic obstructive pulmonary diseases (COPD), asthma, and idiopathic pulmonary fibrosis cause dysbiosis. Dysbiosis occurs when there is a distortion of the microbiome. Generally, microbial immigration, elimination, or cell reproduction are the main factors influencing the respiratory microbiome [23]. Several interactions exist between the respiratory microbiome and other parts of the body. There is the gut-lung and nose-brain axis. The gut-lung axis has been well studied. The relationship here is bidirectional. Such relationship has also been suggested for the nose-brain axis. It is believed to play a role in the modulation of the immune system, metabolic system and development of the nervous system. All these influence the pathophysiology of neurological diseases like Alzheimer’s and Parkinson’s disease [24]. One of the early signs of Parkinson’s disease is loss of smell, and alteration of the nasal microbiota has been implicated as the cause [25, 26].

### The interplay between HMPV and the respiratory microbiome

The respiratory microbiome has different niches, with less abundance in the lungs. The most studied is the bacteriome. The studies of mycobiome and virome are fewer due to the difficulty involved. There are different relationships among members of the bacteriome of the respiratory system. Some of these are pathogenic and are called pathobionts. Examples include *Streptococcal pneumoniae*,* Haemophilus influenza*,* Staphylococcus aureus* and Moraxella catarrhalis. In the study by Sepulveda–Alfaro et al. (2024) [26], he found out that infection with HMPV caused changes in the gut microbiome even when HMPV itself was not isolated. The severity of acute respiratory distress in viral infection also correlates with respiratory microbiota composition [27]. The effect of viral infection causes junctional dysfunction by downregulating E-cadherin production [28]. In addition, infection by and replication of viruses in the epithelial cells causes cell death. Damage to these epithelial cells is a point of entry for pathogenic bacteria due to loss of mucociliary function and direct attachment to the basement membrane [29].

Several mechanisms have been proposed as means by which HMPV disrupts the respiratory microbiome. The suppression of beneficial microorganisms and, by implication, enablement of pathogenic organisms was elucidated by Lalbiaktluang et al. (2023) [30] The alterations in chemokine and cytokine production [31] and increased oxidative stress [32] are other ways it achieves microbiome disruption. HMPV modulates antimicrobial peptide production and function. This impacts the microbiome and correlates with increased susceptibility to allergy and superimposed bacterial infection [33]. A key feature of HMPV infection is increased mucin production [34], which is also found in other viruses [35, 36]. Increased mucin production leads to pockets of hypoxia and increased temperature. This significantly affects organisms that can survive, causing a significant microbiome change [23].

Not only does HMPV impact the microbiome, but the microbiome also has a significant effect on it by modulating its replication and virulence. This was demonstrated by Verkaik et al. (2011) [37], who noticed that the presence of *Streptococcus pneumoniae* enhances the replication of HMPV. When there is abundant S. *pneumoniae* in the respiratory tract, HMPV thrives. Exaggerated inflammatory response has been associated with HMPV infection in the presence of *Haemophilus influenza* [23]. It activates the innate immune responses by infection of the dendritic cells and alveolar macrophages. This leads to cytokine and chemokine productions, in particular, Tumor necrosis factor-alpha (TNF-alpha), Interleukin-6 (IL-6), Interleukin- 8(IL-8), and Interferon -gamma (IFN - gamma) [39]. The Chemokines stimulated are C-C Motif Chemokine Ligand 5 (CCL5) and C-X-C Motif Chemokine Ligand 8 (CXCL8). They both play an important role in recruiting immune cells to the site of infection. The complement system is activated, too, to enhance the clearing of the virus [31]. However, some peculiarities are associated with HMPV infection. These include impaired response to Interferon [40] and markedly elevated IL-6 and TNF-alpha production [31]. The adaptive immune responses to HMPV infection include activation of CD4 + and CD8 + T cells, antibody production, and memory immune cell development. Human metapneumovirus infections have poorly understood effects on CD4 + T cells. However, research shows that HMPV can inhibit CD4 + T cell activation in laboratory settings [41]. When dendritic cells (DCs) infected with HMPV were co-cultured with CD4 + T cells, T cell activation decreased significantly. This decrease may be due to the presence of HMPV’s G and SH proteins. Interestingly, direct contact between DCs and T cells was necessary for this decrease.

**Fig. 3:**
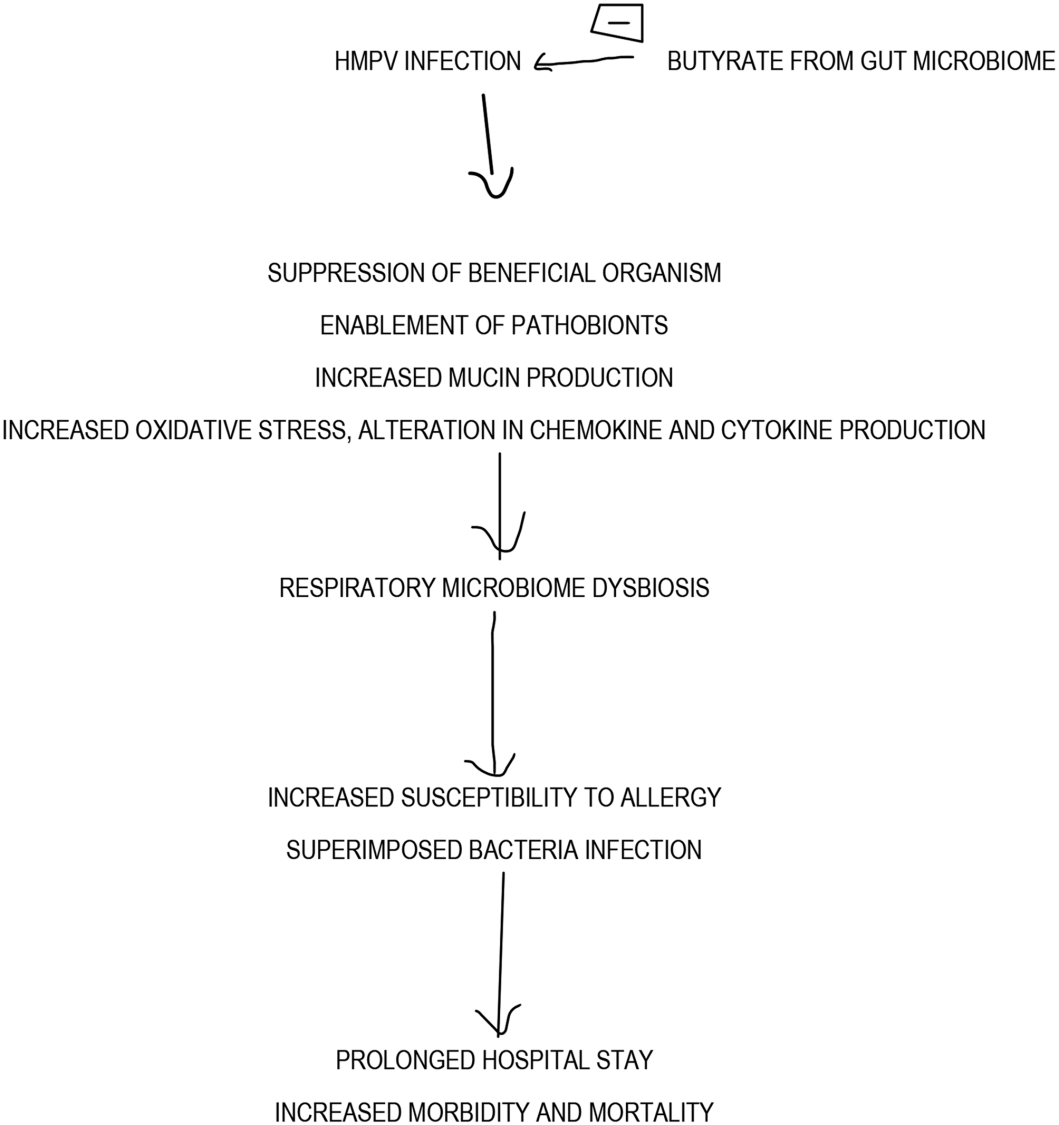
Summary of the effect of HMPV on the Respiratory Microbiome

Identifying antigenic targets on HMPV is crucial for developing effective treatments. Two epitopes from HMPV proteins (N307-315 and M2-181-89) elicited a protective CTL response in mice. Adoptive transfer of CTLs specific to these epitopes protected mice from initial HMPV infection. However, subsequent infections led to a decrease in the protective response [42]. According to Dickson et al. (2016) [23], the presence of Haemophilus influenzae among the respiratory microbiota brings about the increased expression of adhesion molecules like Intercellular cell adhesion molecule − 1( ICAM-1) and Vascular cell adhesion molecule-1 (VCAM-1), improving the attachment of HMPV to the epithelial cells, enhanced production of proinflammatory cytokines like IL-6, IL-17, and TNF- alpha, and suppression of the production of anti-inflammatory cytokine- IL-10. Although Streptococcus pneumoniae stimulates an increase in antibody production targeted at HMPV, it impairs their function and promotes elaborate inflammatory response and tissue damage. This worsens the clinical outcome of HMPV infection. Studies have associated S. pneumoniae, H. influenza, M. catarrhalis and S aureus with increased HMPV severity as earlier mentioned [23]. The link between exacerbations of chronic lung diseases and the severity of HMPV infection may be related to the microbiota. A similar microbiota (that contains S. pneumoniae, H. influenza, M. catarrhalis and S aureus) is seen in patients with lung cancer and may account for HMPV severity in that population. In addition, the presence of Neisseria lactamica and meningitidis in individuals with asthma is linked to severe HMPV infection [23]. In infants, the presence of Ureaplasma urealiticum and Mycoplasma pneumoniae has been correlated with severe HMPV disease. The summary of the impact of HMPV on the respiratory microbiome is shown in Fig. 3.

### HMPV infection and the gut microbiome

Several studies have been done on the gut microbiome, where about 1000 microbial species live [43]. About 80% of the body’s immune cells reside in the gut [44]. This means that the gut’s microbiome and its immune cells have impact on the body’s response to infection. Interestingly, the immune system would not have developed nor matured properly without the gut microbiota. The gut microbiota is acquired through many mechanisms including mode of delivery, feeding, use of antibiotics and the environment. By the time a child is weaned, the gut microbiome has closely resemble the adult’s. The gut microbiota stimulates the development and maturation of the innate immune system through the gut- associated lymphoid tissue. Lower number of natural killer cells and macrophages with poorly developed dendritic and innate lymphoid cells are seen in germ- free mice as compared to conventional mice [45]. The ability of the body to tolerate self antigen is grossly dependent on the gut’s microbiome. This is done by the development and maintenance of T- regs. Specifically, symbiotic bacteria can stimulate the differentiation of TH17 cells, while butyrate, SFA produced by the gut microbiome, can enhance the memory effect of CD8 + cells [46]. Butyrate also inhibit HMPV replication in the lungs [38]. This is not dependent on the presence of the virus in the gut. Infections generally reduce appetite and this can alter the gut microbiota. HMPV infection is not an exemption.

### Viro-bacteria interacton and HMPV infection

HMPV pathogenesis involves attachment of the virus to the epithelial cells and its replication within the cells lead to destruction of the cells and exposure of the underlying structures to bacteria attachment. The respiratory epithelial lining is continuous and the cells separated by tight junctions, thereby preventing bacteria infection. Loss of the epithelial cells remove this protective barrier. In addition, HMPV induces increased mucin production, causing dysbiosis in the respiratory tract with overgrowth of pathogenic bacteria. HMPV, like most viral infection can be self-limiting, however, super-imposed bacteria infections worsen the prognosis, leading to pneumonia. This not only prolong hospital stay, it also increases morbidity and mortality. Other mechanisms involved in viro-bacteria interaction of HMPV includes disruption of the immune system that should control bacteria infection [47].

### Molecular diagnosis of HMPV

Before the advent of molecular techniques, microscopy, culture and sensitivity testing were used, this was one of the reasons why the lung was believed to be sterile, as obtaining pure samples and growing all the constituents of the microbiome was practically impossible. The need for rapid diagnostic methods became apparent during the COVID-19 outbreak, and they have revolutionized the detection of infections, especially those with the potential to spread fast and cause death in many countries across borders and territories. Sample collection method depends on location along the respiratory tract. Swabs are sufficient for the upper respiratory tract while more invasive ones like tracheal aspirate or bronchoalveolar lavage can be used for lower respiratory sites. Specific methods for HMPV include:

### Reverse transcriptase polymerase chain reactions (RT-PCR)

As HMPV is an RNA virus, cDNA needs to be made using reverse transcriptase before proceeding to other forms of PCRs. Polymerase chain reaction has become the mainstay in the diagnosis of infectious disease as the genome of the pathogen can be obtained. This is important because of the similar ways by which infectious organisms especially viruses present clinically. Sugimoto et al. (2023) [48] modified this method to detect the two subgroups of HMPV. It is difficult to differentiate infection caused by RSV from that of HMPV without nucleic acid amplification tests. Other methods used in diagnosis include:

### Quantitative polymerase chain reaction (qPCR)

The importance of real-time PCR cannot be overemphasized in making diagnoses and monitoring treatment. This gives the quantity of the virus present, which can be correlated with disease severity (Sugimoto et al., 2023) [48].

### Multiplex PCR

Clinical presentation of respiratory viral infections cannot be used to make accurate diagnosis because many respiratory viral infections present in similar ways as noted for RSV, influenza and HMPV. Multiplex PCR becomes handy in identifying the presence of one or more of them as the cause of the presentation [49]. Multiplex PCR involves a panel of PCRs where multiple related organisms can be amplified at once. Coinfection with two or more viruses can be detected with this.

### Loop-mediated isothermal amplification (LAMP)

This has been developed to make rapid and sensitive diagnoses in the field [50]. Here, specialized polymerase and sets of primers are used in amplification at a constant temperature of around 65 °C. In 2023, Jiao et al. developed a Reverse transcriptase-mediated isothermal amplification fluorescence assay for HMPV that operates at 39 degrees Celsius and has a minimum detection limit of 10^2 copies per microlitre [51]. It combines real-time, reverse transcription, and fluorescence assay at a single temperature.

### Clustered regularly interspaced short palindromic repeat– CRISPR-associated protein (CRISPR- Cas12a) 

CRISPR-Cas system was imported from the prokaryotes, and has been used successfully in therapy [52]. CRISPR- Cas system is a gene editing methods employed in many fields including treatment of genetic diseases like sickle cell anemia. Guide RNA leads the system to the point where the snips are made in the genome. the ability to particularly target a point on the genetic material makes it useful in diagnosis too. The conserved region of HMPV can be targeted and when added to other methods, can enhance the diagnosis of HMPV. CRISPR- Cas 12a (CRISPR- associated protein 12a) is being combined with other methods like Loop-mediated isothermal amplification (LAMP), Recombinase Aided Amplification (RAA), and Recombinase Polymerase Amplification (RPA). This also reduced the limit of detection to < 700 copies /mL [52].

**Fig. 4:**
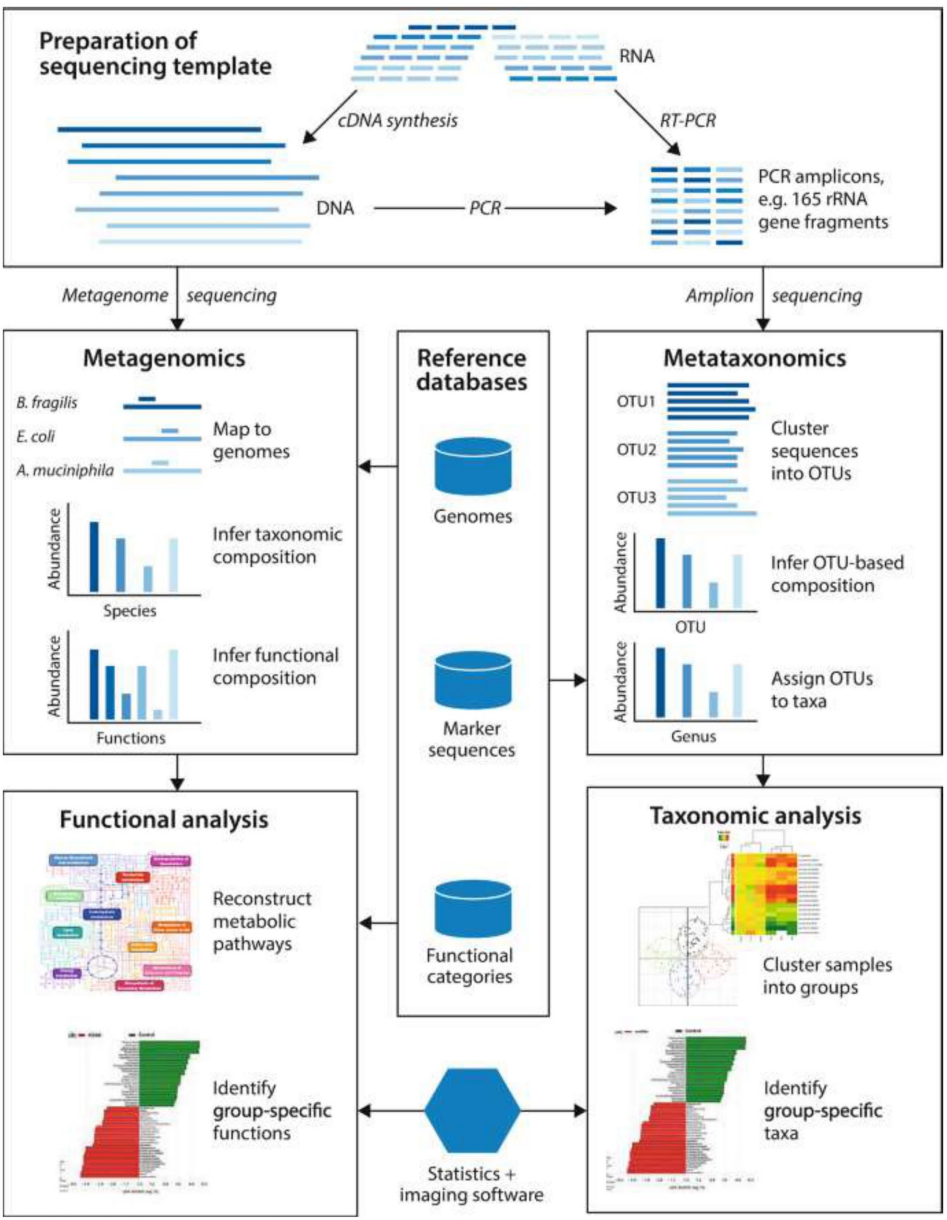
Molecular Microbiome Analysis [55]

Sequencing is needed afterwards. Considering the amount of data needed to be generated from the microbiome study, Sanger’s sequencing methods can be used, but it will take a long time before the results are obtained. This emphasizes the importance of high through-put sequencing like Next- generation Sequencing. The data obtained are analyzed with different bioinformatic tools to be able to make meaningful conclusions. These include data from genomic, epigenomic, transcriptomic, proteomic and metabolomic studies. Integrating the multi-omics findings of infectious diseases and the host-microbiome relationship has enabled the identification of potential markers of susceptibility and poor prognosis [53]. In 2023, Mick et al. [54]. demonstrated that accurate lower respiratory tract infection diagnosis and identification of pathogens in critically ill children was achievable with a metagenomics study that integrated host, microbiome and pathogen characteristics. Figure 4 describes the molecular microbiome analysis.

### Clinical implications and future directions

There is currently no vaccine for HMPV nor a direct-acting antiviral, so microbiota modification is a promising intervention area with few adverse effects. Probiotics are live beneficial organisms that alter the microbiota to increase resistance to invading pathogens and prevent pathobionts transformation. Other effects they can have include modulation of the immune system, production of micronutrients and promotion of general well-being [56]. International Scientific Association for Probiotics and Prebiotics (ISAPP), in 2016, defined prebiotic as a substrate selectively utilized by host microorganisms, conferring a health benefit. Synbiotics are a combination of probiotics and prebiotics with potential immunomodulating and intestinal flora-restoring activity. The primary mechanism by which this act is carried out is by influencing and modulating the microbiota. Examples of probiotics include L*actobacillus* spp *(acidophilus*,* rhamnosus*,* fermentum*,* johnsonii*,* lactose and reuteri*), bifidobacterium spp (*breve*,* infants*,* longum*,* bifidum*,* lactose*,* thermophilum)*, *Bacillus coagulans*,* Streptococcus thermophillus*, *Enterococcus faecium* and *Saccharomyces cerevisae* [57].

Prebiotics are compounds of carbohydrates or oligosaccharides. They are lactulose, xylooligosaccharides, galactooligosaccharides, inulin and its derivatives [49].

When dead organisms with or without their products of metabolism are administered, they are called postbiotics (Vinderola et al., 2023). *Akkermansia muciniphila* has anti-inflammatory effects even when dead. It is being marketed as a postbiotic [58].

In 2021, Darbandi et al. [59] conducted a systematic review looking at the effects of probiotics on respiratory tract infections. A total of 24 studies out of 27 show that probiotics boost immune response and assist the body in fighting viral infections, and the ones that focused on the impact of probiotics and prebiotics on vaccine performance revealed improved performance when given together. Microbiome-based therapy was explored in inflammatory conditions of the GIT, such as faecal microbiota transplantation, where the healthy donor microbes are transplanted to patients with recurrent *Clostridium difficile* infection [60]. Same can also be explored for respiratory diseases, primarily via the gut-lung axis. Understanding the respiratory microbiome profile in health and disease can bring about targeted care, improvement in disease conditions, reduction of cost of care and limitation of adverse effects arising from using drugs that will not work or that may worsen the clinical state of the patient. In addition, chronic health conditions can be adequately managed using the knowledge obtained by studying probiotics, prebiotics, synbiotics and postbiotics, to reduce the frequency of exacerbations and retard progression [61].

### The microbiota and vaccine

The microbiota impacts orally-administered vaccines as they come in contact with them in the GIT. This is done by modulation of the immune system, and as the microbiota are the responders to orally administered vaccines, dysbiosis can affect response to them [62, 63]. The microbiota also impact the absorption and distribution of vaccines by altering the tight junctions [64]. On the other hand, the vaccine affects the microbiota. This was established by Medeiros et al. (2022) [65] when they demonstrated significant improvements in the health of the intestinal microbiota after oral polio vaccination. The microbiota influences the parenterally administered vaccines too, through regulation of the immune system, as shown by Ng et al. (2023) [66] and Ray et al. (2023) [67], using COVID-19 vaccines as examples.

Edible vaccines are being developed to combat the challenges encountered in producing, storing and distributing vaccines. They rely significantly on the microbiota of the GIT [68]. These avenues can be explored when developing vaccines against HMPV.

## Future research needs

Though there was an increase in the cases of HMPV in China, but thankfully, none has been reported on our continent so far. However, absence of reports of HMPV in Africa does not preclude adequate preparation, considering the experience of COVID-19. Considering the advancements made in the world of technology, that has enabled us to understand that the lung is not sterile, as previously concluded, and that many interactions occur between the virus and the microbiome, as well as between the host and the virus through the microbiota, much still needs to be done to bring the knowledge to the bedside for precision medicine and improvements in the lives of patients. Noninvasive methods of sampling of the lower respiratory tract needs to be developed. In addition, we need to find ways of delivering the probiotics and prebiotics directly into the respiratory system, especially the lungs, without worsening the patients’ clinical conditions. More studies on the lung microbiome are needed in the healthy. This can be achieved when there are ways to take samples noninvasively. Personalized medicine can be achieved with robust research on pharmacogenomics and pharmacomicrobiomics in individuals. Studies on edible vaccines are still in the early stages; more needs to be done to achieve that, considering the microbe’s inherent ability to mutate rapidly.

## Conclusion and Recommendations

The intersection between HMPV and the respiratory microbiome and the severity and prognosis of the infection has been explored in the preceding segments. The spectrum of HMPV presentation, ranging from mild to severe, can be hinged upon the respiratory microbiome, which, in turn, is formed based on factors ranging from the mode of delivery to immunization status, exposure to daycare, cigarette smoke, and even genetics. The presence or absence of chronic respiratory diseases also shapes the microbiota and influences the viral-bacterial or bacterial-bacterial interactions. Thanks to the advent of cutting-edge technologies like next-generation sequencing, the previously assumed sterile lung is now recognized to be home to different populations of microbes including bacteria, fungi, and phages. These all affect the infection and response to HMPV. Modifying the respiratory microbiota indirectly through the gut-lung axis and possibly directly by administering probiotics, prebiotics, synbiotics, and postbiotics are ways by which HMPV can be managed to limit the adverse effects of medications currently being used and bring about personalized medical care.

### Recommendations

The lung microbiome analysis can enhance the diagnosis of HMPV infection by the shift in the microbiome. The shift can be changes in alpha and beta diversity. Identifying the patterns of bacterial infection that include pathobionts like S. pneumoniae, H influenza, M. catarrhalis and S. aureus, especially in patients with chronic lung diseases, and U. urealyticum in infants, can be pointers to severe HMPV infection. Considering the potential for diagnoses and management of respiratory viral illnesses like HMPV, based on the microbiome, it is important that more researches are done to study the mycobiome and the virome, as the ones done are skewed towards the bacteriome. Systematic review and meta-analysis are also needed to verify the relationship between HMPV and the respiratory microbiome. HMPV infection leads to microbiome dysbiosis as highlighted above. As the respiratory and gut microbiome affects drug metabolism either directly, by altering the pharmacokinetics of the drugs, or indirectly through production of short-chain fatty acid like butyrate, understanding both will impact the therapeutic effects of known antivirals -like ribavirin and subsequent ones that will be HMPV- specific. The current knowledge on the respiratory microbiome should be expanded to include healthy individuals of African descent.

To be able to achieve this, noninvasive sampling methods are needed to minimize harm to the research participants. The researches should integrate the omics for holistic and personalized results.

Funding is an important factor in conducting standard researches, not to talk of the ones that will involve a significant proportion of the populace, as needed in this context. Such large amount of money cannot come from the pockets of the researchers. The interest and commitment of governments and philanthropists that understands the impact of high-quality researches must be obtained. When such are done, the policymakers must be informed so that the outcome is brought to the bedside of the patients.

Screening for HMPV and other respiratory viral infections should be ongoing, in addition to other surveillance approaches needed to nip HMPV in the bud anytime it surfaces in Africa in general, and in Nigeria in particular.

Lastly, while we await the funds, researches, the change in policy, and the outcome of our surveillance reports, let us not discard the lessons learnt during COVID-19 infection such as hand- washing, social distancing and other general infection prevention and control methods, as these may be our saving grace, protecting us from not only HMPV but other known and yet -to- be- known respiratory viruses.

The limitations of this review include the following:

Publication Date Restriction- We limited our search to studies published before March 2025, therefore, very recent developments may not be fully represented in this article.

Language bias- All the reviewed articles are in English language publications, potentially excluding relevant studies published in other languages.

Reliance on published data- This review is completely dependent on published data. Significant information from unpublished data could not be accessed.

## Data Availability

No datasets were generated or analysed during the current study.
